# Prognostic significance of prognostic nutritional index in patients with head and neck squamous cell carcinoma

**DOI:** 10.3389/fimmu.2025.1597965

**Published:** 2025-08-27

**Authors:** Yongping Wang, Jie Wang, Binbin Xiao, Yuqing Wang, Fu Huang, Yang Jiang, Tianyi Liu

**Affiliations:** Department of otorhinolaryngology, Renmin Hospital of Wuhan University, Wuhan, Hubei, China

**Keywords:** prognostic nutritional index, head and neck squamous cell carcinoma, prognosis, overall survival (OS), disease-free survival (DFS)

## Abstract

**Objective:**

This study aimed to investigate the relationship between prognostic nutritional index (PNI) and prognosis in patients with head and neck squamous cell carcinoma (HNSCC).

**Methods:**

A systematic review was conducted across three major databases—Embase, PubMed, and the Cochrane Library—to identify studies examining the association between PNI and outcomes in HNSCC patients. The search included all records from database inception through January 20, 2025. Outcomes assessed included hazard ratios (HRs) for overall survival (OS), cancer-specific survival (CSS), disease-free survival (DFS), and progression-free survival (PFS), as well as odds ratios (ORs) for objective response rate (ORR) and disease control rate (DCR).

**Results:**

A total of 27 articles involving 4,400 patients were included. Patients with low PNI had significantly shorter OS (HR: 2.42, 95% CI: 2.15–2.73, *p* < 0.001), CSS (HR: 2.05, 95% CI: 1.09–3.84, *p* = 0.026), DFS (HR: 1.89, 95% CI: 1.58–2.27, *p* < 0.001), and PFS (HR: 2.23, 95% CI: 1.90–2.62, *p* < 0.001) compared to those with high PNI. Additionally, low PNI was associated with lower ORR (OR: 0.40, 95% CI: 0.22–0.73, *p* = 0.002) and DCR (OR: 0.30, 95% CI: 0.17–0.53, *p* < 0.001). Subgroup analyses confirmed consistent associations between PNI and OS, DFS, and PFS across different Cox models, cancer types, treatment modalities (immune checkpoint inhibitors and surgery), countries, and PNI cut-off values.

**Clinical trial registration:**

This study underscores the prognostic significance of PNI in predicting survival outcomes and treatment responses in HNSCC patients. The findings highlight the importance of incorporating PNI into routine prognostic assessments to improve clinical decision-making and patient management in HNSCC.

## 1 Introduction

Head and neck squamous cell carcinoma (HNSCC) encompasses a diverse group of tumors originating from various anatomical sites, including the oral cavity, oropharynx, hypopharynx, and larynx (1, 2). Therapeutic approaches for HNSCC include surgical resection, radiotherapy, chemotherapy, targeted molecular therapies, and immune checkpoint inhibitors (ICIs) (3). Over the past decade, advances in treatment strategies have significantly improved therapeutic outcomes. However, predicting prognosis remains a major challenge for head and neck surgeons. To optimize treatment strategies, there is an urgent need to identify reliable biomarkers that can more accurately predict both prognosis and treatment response (4, 5).

The prognostic nutritional index (PNI) is calculated based on serum albumin concentration and lymphocyte count. Serum albumin is a recognized biomarker of nutritional status and has been linked to comorbidities and cancer prognosis (6, 7). Lymphocytes, as key mediators of cell-mediated immunity, play a critical role in suppressing cancer cell proliferation and invasion (8). As a result, PNI provides an integrated measure of both the nutritional and immunological health of a patient. Initially introduced as a predictor of postoperative complications in gastrointestinal cancer patients (9), recent research has established its relevance in predicting clinical outcomes across various cancer types (10–13).

The predictive value of PNI in HNSCC patients, however, remains controversial. For instance, studies by Abe et al., Matsumura et al., and Miyamoto et al. reported that HNSCC patients with high PNI levels had longer overall survival (OS) (14–16). In contrast, studies by Ikeguchi et al., *Song* et al., and *Tada* et al. suggested that PNI levels were not significantly correlated with prognosis (17–19).

This study aims to resolve the controversy by systematically synthesizing all available evidence, thereby enhancing our understanding of the clinical significance of PNI in predicting prognosis for HNSCC patients. To the best of our knowledge, this is the first pooled analysis to comprehensively evaluate the role of PNI in predicting both prognosis and treatment response in patients with HNSCC.

## 2 Methods

### 2.1 Search strategy

An electronic search was initiated on January 20, 2025, across major bibliographic databases, including EMBASE, PubMed, and the Cochrane Library. The search used predefined terms such as “Squamous Cell Carcinoma of Head and Neck” [Mesh], “Oral Tongue Squamous Cell Carcinoma,” “Hypopharyngeal Squamous Cell Carcinoma,” “Oropharyngeal Squamous Cell Carcinoma,” “Laryngeal Squamous Cell Carcinoma,” and “prognostic nutritional index,” covering all relevant domains. The search was limited to human studies published in English. A detailed description of the search strategy is provided in **Supplementary Material 1**. Additionally, grey literature was sourced from Google Scholar, and reference lists of relevant studies were manually reviewed. Following Cochrane collaboration guidelines, findings from both manual and electronic searches were compiled in Covidence software for data management.

### 2.2 Inclusion and exclusion criteria

We established the following inclusion criteria for article selection: (i) studies involving patients diagnosed with HNSCC; (ii) studies assessing the prognostic significance of baseline PNI; and (iii) studies reporting at least one of the following clinical outcomes: OS, progression-free survival (PFS), disease-free survival (DFS), cancer-specific survival (CSS), objective response rate (ORR), or disease control rate (DCR). The exclusion criteria were: (i) studies based on animal models, literature reviews, case reports, or conference abstracts; (ii) studies lacking hazard ratios (HRs) or odds ratios (ORs) for outcome evaluation, either from the main text or published data. In cases where multiple studies included overlapping patient cohorts, preference was given to those with more comprehensive data and robust methodological quality.

### 2.3 Data extraction and quality assessment

During data extraction, we systematically collected key details, including authorship, publication year, study period, geographic location, cancer types, treatment modalities, sample size, demographic information (age and gender), and PNI cut-off values. The primary data sources for HRs, ORs, and their respective 95% confidence intervals (CIs) were multivariate analyses. When these were unavailable, data were either derived from univariate analyses or extracted from survival plots using Engauge Digitizer software. The quality of the included observational studies was assessed using the Newcastle-Ottawa Scale (NOS), with studies scoring six or above considered of high quality. The nine-point NOS criteria evaluate areas such as patient selection, study comparability, and outcome measurement. All stages of the process, from literature retrieval and screening to data extraction and quality evaluation, were independently performed by two researchers, with discrepancies resolved through consultation with the senior author.

### 2.4 Statistical methods

Statistical analyses were conducted using Stata 18.0, with results visualized through forest plots. Heterogeneity was assessed using Cochran’s Q test and I² statistics, with significant heterogeneity defined as a *p*-value < 0.1 and I² > 50%. In cases of substantial heterogeneity, the DerSimonian-Laird random-effects model was employed, while the Inverse Variance fixed-effects model was used otherwise (20). To evaluate the potential for publication bias, we used funnel plots when the number of included studies for a specific outcome was ≥12, in accordance with PRISMA and MOOSE guidelines. For outcomes with fewer than 12 studies, the statistical power of funnel plot asymmetry tests is limited. Therefore, we applied Begg’s tests to assess publication bias in these cases (21). The robustness of the findings was tested through sensitivity analyses by systematically excluding individual studies (22). Additionally, subgroup analyses were performed, focusing on different Cox models, cancer types, treatment modalities, countries, and PNI cut-off values. A two-tailed *p*-value < 0.05 was considered statistically significant.

## 3 Results

### 3.1 Search results and included studies

**Figure 1:**
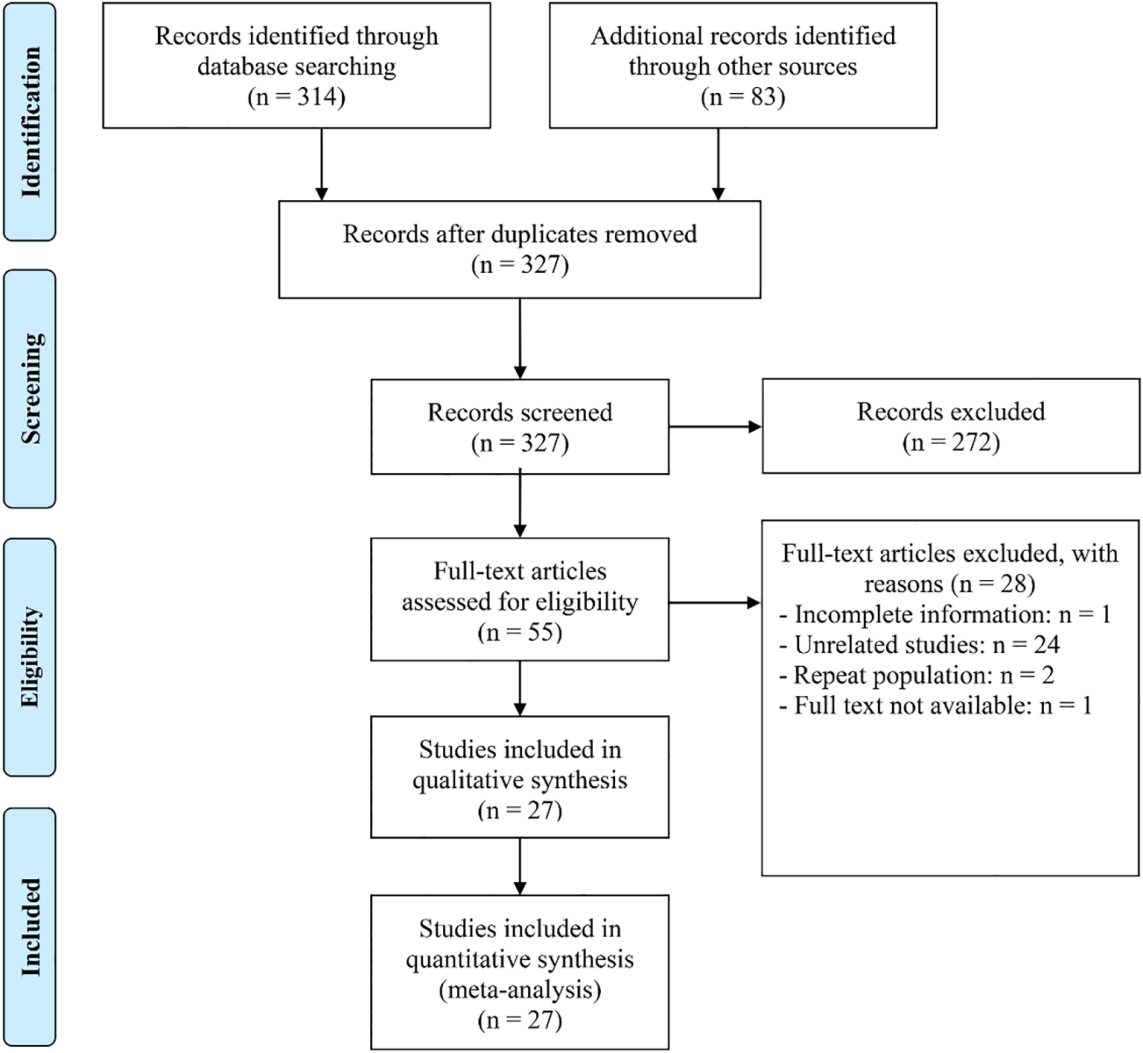
The flow diagram of identifying eligible studies.

The initial search strategy, combined with manual review, identified 397 potentially relevant articles. After removing 70 duplicates, 272 articles were excluded for failing to meet the inclusion criteria based on their titles and abstracts. A thorough evaluation of the remaining 55 full-text articles led to the exclusion of 28, as they did not fulfill the established criteria. As a result, 27 articles with 29 studies were ultimately deemed eligible for inclusion (**Figure 1**) (1, 4, 14–19, 23–41).

### 3.2 Study characteristics

**Table 1** summarizes the key characteristics of the studies included in this analysis. The cohort comprised 4,400 patients, of whom 73.65% were male. Sample sizes ranged from 42 to 661 individuals. Among the studies, 14 were conducted in Japan, seven in China, and two in the United States. Additionally, one study was conducted in Canada, one in Hungary, one in Italy, one in Korea, one in Tottori, and one in the USA. Treatment modalities varied: 14 studies involved surgical treatment, 6 studies used ICIs, 3 studies utilized chemoradiotherapy, and 3 studies applied comprehensive therapy. All studies were retrospective, with NOS scores ranging from 6 to 8, indicating a low risk of bias (**Table 1**).

**Table 1:** Main characteristics of the studies included.

Study	Study period	Country	Sample size	Age	Gender (male/female)	Treatment	Cut‐point	NOS
Go et al., 2024	01/2009-12/2019	Korea	101	67.87^a^	96/5	Surgery	48.27	7
Ohyama et al., 2024	01/2014-01/2021	Japan	146	69.90^a^	67/79	Surgery	51.40	8
Matsumura et al., 2024	05/2017-01/2021	Japan	65	65 (26–82)^b^	50/15	ICIs	39.10	7
Tomasoni et al., 2023	03/2004-06/2018	Italy	542	67 (60–75)^c^	392/150	Surgery	49.60	8
Tanaka et al., 2023	04/2017-12/2020	Japan	42	61 (26–81)^d^	36/6	ICIs	42.00	6
Oka et al., 2023	01/2010-12/2018	Japan	124	63 (34–83)^b^	103/21	Comprehensive therapy	41.00	7
Miyamoto et al., 2023	2017-2022	Japan	106	68 (21–88)^b^	83/23	ICIs	41.9	7
Hernando-Calvo et al., 2023	11/2014-03/2021	Canada	100	63 (22–84)^b^	82/18	ICIs	40	7
Yoshimura et al., 2022	01/2009-12/2015	Japan	112	68 (59–77)^c^	69/43	Surgery	50.61	8
Kubota et al., 2022	2005-2017	Japan	183	66 (26–93)^b^	103/80	Comprehensive therapy	52.40	7
Fang et al., 2022	01/2007-12/2017	China	360	97/267^f^	325/35	Surgery	51.75	8
Watabe et al., 2021	–	Japan	110	68 (58–76)^c^	61/49	Surgery	52.44	7
Guller et al., 2021	2014-2020	USA	99	64 (57–70)^c^	86/13	ICIs	45.00	6
Abe et al., 2021	01/2008-06/2019	Japan	102	65.6 ± 9.8	73/29	Surgery	42.93	7
Yoshida et al., 2020	01/2004-12/2011	Japan	47	79 (45–90)^b^	23/24	Chemoradiotherapy	42.69	6
Wu et al., 2020 (T)	04/2011-12/2018	China	166	91/75^e^	89/77	Surgery	47.40	7
Wu et al., 2020 (V)	01/2004-12/2016	China	167	109/58^e^	86/81	Surgery	47.40	7
Ye et al., 2018	03/2006-08/2016	China	123	57 (32–87)^b^	121/2	Surgery	52.00	6
Bruixola et al., 2018 (T)	05/2010-05/2016	Spain	50	55 (41–59)^d^	42/8	Chemoradiotherapy	45.00	7
Bruixola et al., 2018 (V)	05/2010-05/2016	Spain	95	60 (43–77)^b^	90/5	Chemoradiotherapy	45.00	7
Ikeguchi et al., 2016	2004-2014	Tottori	59	68.7 ± 9.5	57/2	Surgery	40.00	8
Chiu et al., 2024	01/2017-12/2022	China	144	59 (28–91)^b^	131/13	ICIs	45.00	7
Song et al., 2024	04/2014-12/2021	China	58	54 (42–64)c	40/18	Surgery	49.30	7
Tada et al., 2021	–	Japan	44	66 (47–86)^b^	–	–	49.43	7
Sakai et al., 2023	06/2017-06/2022	Japan	51	66 (47–83)^b^	48/3	Chemoradiotherapy	40.00	6
Uri et al., 2024	2014-2023	Hungarian	661	–	528/133	–	–	7
Yamagata et al., 2022	2013-2017	Japan	155	–	95/60	Comprehensive therapy	49.30	7
Li et al., 2024	01/2015-04/2018	China	262	159/103^e^	176/86	Surgery	45.50	8
Fukuzawa et al., 2024	01/2011-12/2020	Japan	126	67 (29–92)^b^	69/57	Surgery	51.05	7

^a^mean, ^b^median (range), ^c^median (IQR), ^d^mean (range), ^e^Age ≥ 60 years *vs.* < 60 years, ^f^Age ≥ 65 years *vs.* < 65 years. ICIs, immune checkpoint inhibitors; HNSCC, head and neck squamous cell carcinoma.

### 3.3 Baseline prognostic nutritional index and overall survival and cancer-specific survival

**Figure 2:**
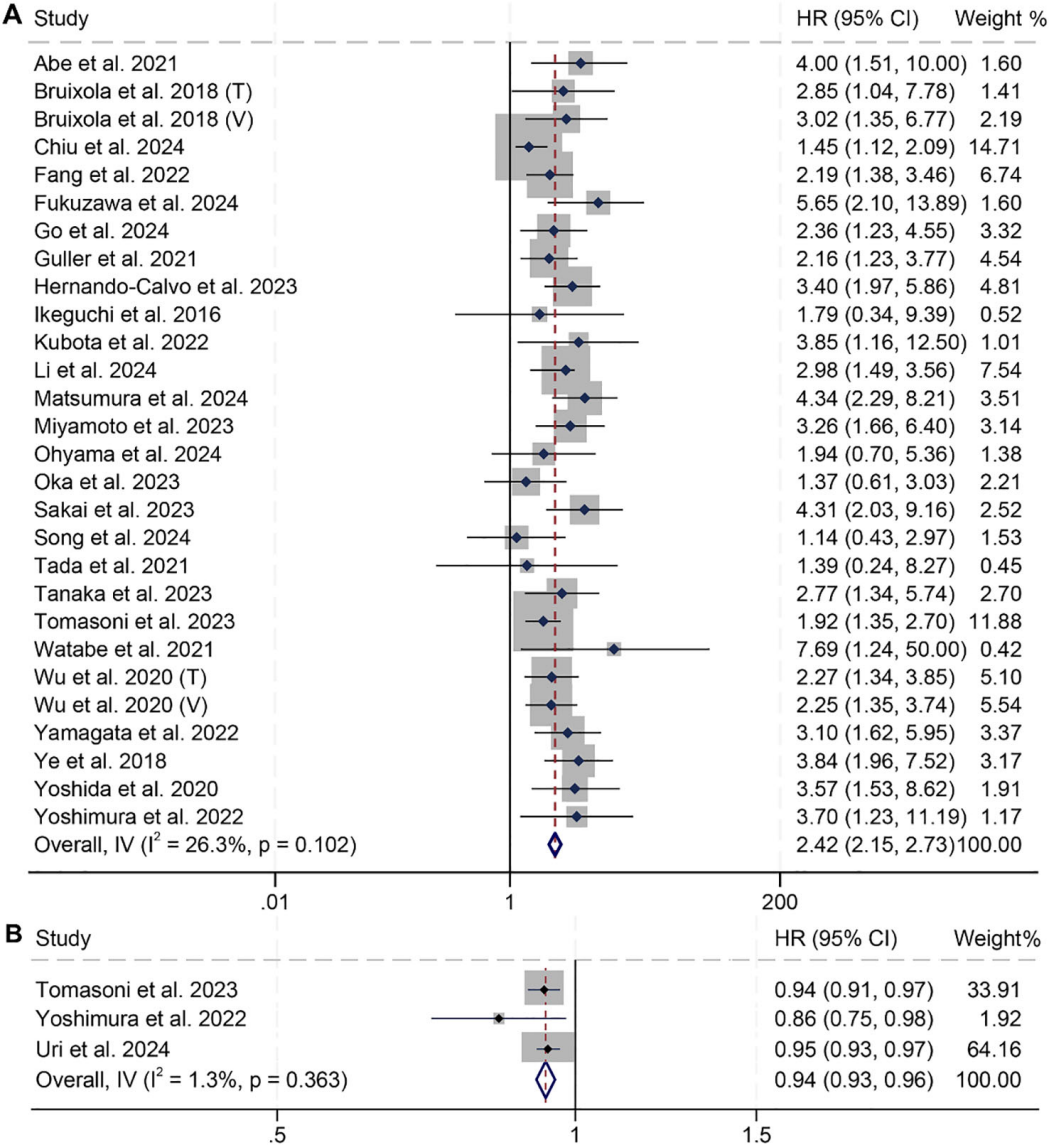
Forest plots showing the association between prognostic nutritional index (PNI) and overall survival (OS). **(A)** PNI analyzed as a binary variable (high vs. low); **(B)** PNI analyzed as a continuous variable (per unit increase). HR, hazard ratio; CI, confidence interval.

In this study, we included 28 studies comprising a total of 3,739 cancer patients to examine the impact of high and low PNI on OS in patients with HNSCC. The analysis revealed that patients with low PNI had significantly shorter OS (HR: 2.42, 95% CI: 2.15–2.73, *p* < 0.001, **Figure 2A**) compared to those with high PNI. No significant heterogeneity across studies was found, as indicated by Cochran’s Q test and I² statistics (I² = 26.3%, *p* = 0.102). Therefore, a fixed-effects model was applied. In addition, three studies treated PNI as a continuous variable and found that higher PNI was associated with longer OS in patients (I² = 1.3%, *p* = 0.363; HR: 0.94, 95% CI: 0.93–0.96, *p* < 0.001, **Figure 2B**).

Subgroup analyses confirmed that the association between PNI and OS was consistent across subgroups with different Cox models, treatment modalities, countries, and PNI cut-off values (**Table 2**).

**Table 2:** Subgroup analysis of the association between prognostic nutritional index and overall survival in patients with head and neck squamous cell carcinoma.

Variable	Included studies	Test of association			Test of heterogeneity		
		HR	95%CI	*p*-value	Modal	I^2^	*p*-value
Cox model							
Multivariate analysis	19	2.60	2.24-3.02	*p* < 0.001	R	0	*p* = 0.492
Univariate analysis	9	2.40	1.73-3.33	*p* < 0.001	R	51.9%	*p* = 0.034
Treatment							
Immune checkpoint inhibitors	6	2.61	1.76-3.87	*p* < 0.001	R	67.5%	*p* = 0.009
Chemoradiotherapy	4	3.48	2.28-5.30	*p* < 0.001	R	0	*p* = 0.899
Surgery	14	2.45	2.06-2.91	*p* < 0.001	R	4.5%	*p* = 0.402
Comprehensive therapy	3	2.43	1.33-4.44	*p* = 0.004	R	34.7%	*p* = 0.216
Country							
Japan	14	3.28	2.60-4.13	*p* < 0.001	R	0	*p* = 0.668
Spain	2	2.95	1.57-5.54	*p* = 0.001	R	0	*p* = 0.928
China	7	2.17	1.64-2.87	*p* < 0.001	R	52.9%	*p* = 0.047
Other	5	2.25	1.77-2.85	*p* < 0.001	R	0	*p* = 0.540
Cut-off							
39-43	9	3.23	2.51-4.14	*p* < 0.001	R	0	*p* = 0.570
45	5	2.23	1.55-3.22	*p* < 0.001	R	55.0%	*p* = 0.064
47-53	14	2.38	2.00-2.83	*p* < 0.001	R	0	*p* = 0.449

HR, hazard ratio; CL, confidence interval; R, random-effect model.

**Figure 3:**
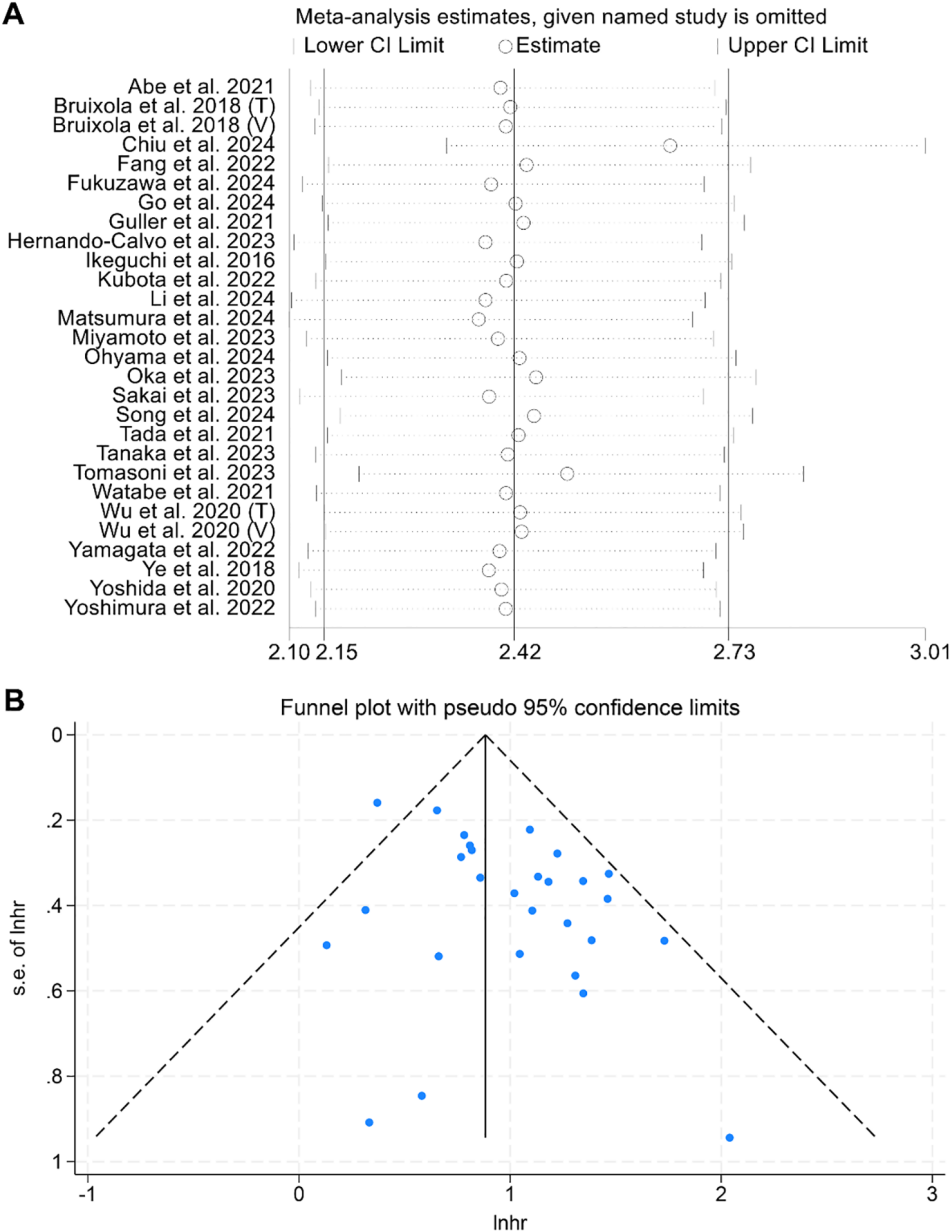
**(A)** Sensitivity analysis of the association between prognostic nutritional index (PNI) and overall survival (OS), based on sequential exclusion of each included study. **(B)** Funnel plot assessing publication bias in the analysis of PNI and OS. HR, hazard ratio; CI, confidence interval.

Sensitivity analysis, which systematically excluded each study, demonstrated that the pooled HRs for OS remained stable and robust (**Figure 3A**). Assessments of publication bias using funnel plots and Begg’s test showed no significant bias (Begg’s test: *p* = 0.441, **Figure 3B**).

We also found that HNSCC patients with low PNI had shorter CSS compared to those with high PNI (Binary variables: HR: 2.05, 95% CI: 1.09–3.84, *p* = 0.026; Continuous variables: HR: 0.94, 95% CI: 0.92–0.97, *p* < 0.001) (**Supplementary Figures S1A, B**).

### 3.4 Pretreatment prognostic nutritional index and disease-free survival and progression-free survival

**Figure 4:**
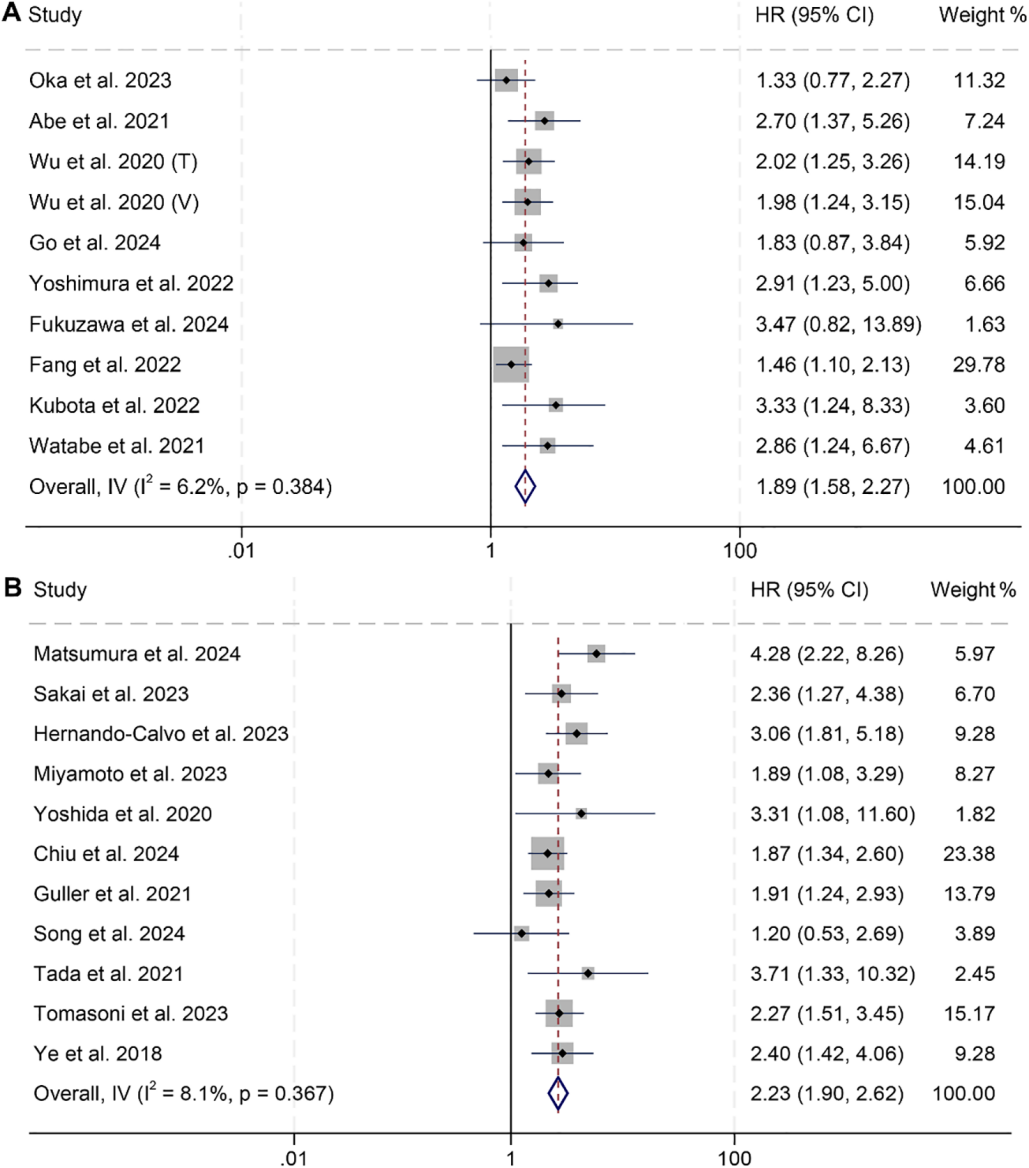
Forest plots showing the association between prognostic nutritional index (PNI) and survival outcomes: **(A)** Disease-free survival (DFS); **(B)** Progression-free survival (PFS). Both panels represent analyses using PNI as a binary variable (high vs. low). HR, hazard ratio; CI, confidence interval.

A total of 10 studies involving 1,551 patients and 11 studies with 1,379 patients examined the predictive value of PNI on DFS and PFS in HNSCC patients, respectively. The findings revealed that cancer patients with low PNI had significantly poorer DFS (I² = 6.2%, *p* = 0.384; HR: 1.89, 95% CI: 1.58–2.27, *p* < 0.001, **Figure 4A**) and PFS (I² = 8.1%, *p* = 0.367; HR: 2.23, 95% CI: 1.90–2.62, *p* < 0.001, **Figure 4B**). Subgroup analyses further demonstrated that low PNI was significantly associated with worse DFS and PFS across various subgroups, with detailed results presented in **Tables 3**, **4**.

**Table 3:** Subgroup analysis of the association between prognostic nutritional index and disease-free survival in patients with head and neck squamous cell carcinoma.

Variable	Included studies	Test of association			Test of heterogeneity		
		HR	95%CI	*p*-value	Modal	I^2^	*p*-value
Cox model							
Multivariate analysis	7	1.90	1.55-2.32	*p* < 0.001	F	0	*p* = 0.444
Univariate analysis	3	1.88	1.25-2.83	*p* = 0.002	F	47.1%	*p* = 0.151
Treatment							
Surgery	8	1.94	1.59-2.36	*p* < 0.001	F	0	*p* = 0.475
Comprehensive therapy	2	1.66	1.04-2.66	*p* = 0.034	F	62.8%	*p* = 0.101
Country							
Japan	6	2.72	1.67-3.08	*p* < 0.001	F	13.1%	*p* = 0.331
China	3	1.70	1.35-2.16	*p* < 0.001	F	0	*p* = 0.429
Korea	1	1.83	0.87-3.84	*p* = 0.113	–	–	*-*
Cut-off							
39-43	2	1.76	1.15-2.67	*p* = 0.009	F	61.3%	*p* = 0.108
47-53	8	1.93	1.58-2.35	*p* < 0.001	F	0	*p* = 0.443

HR, hazard ratio; CL, confidence interval; F, fixed-effect model.

**Table 4:** Subgroup analysis of the association between prognostic nutritional index and progression-free survival in patients with head and neck squamous cell carcinoma.

Variable	Included studies	Test of association			Test of heterogeneity		
		HR	95%CI	*p*-value	Modal	I^2^	*p*-value
Cox model							
Multivariate analysis	4	2.44	1.89-3.16	*p* < 0.001	F	19.9%	*p* = 0.290
Univariate analysis	7	2.11	1.72-2.59	*p* < 0.001	F	5.7%	*p* = 0.384
Treatment							
ICI	5	2.20	1.79-2.70	*p* < 0.001	F	43.9%	*p* = 0.129
Surgery	3	2.12	1.57-2.86	*p* < 0.001	F	9.6%	*p* = 0.331
Chemoradiotherapy	2	2.54	1.47-4.39	*p* = 0.001	F	0	*p* = 0.621
Country							
Japan	5	2.71	1.97-3.72	*p* < 0.001	F	3.2%	*p* = 0.388
China	3	1.90	1.46-2.47	*p* < 0.001	F	0.1%	*p* = 0.367
Other	3	2.29	1.77-2.97	*p* < 0.001	F	0	*p* = 0.393
Cut-off							
39-43	5	7.74	2.06-3.63	*p* < 0.001	F	0	*p* = 0.410
45	2	1.88	1.45-2.45	*p* < 0.001	F	0	*p* = 0.941
47-53	4	2.22	1.66-2.96	*p* < 0.001	F	8.2%	*p* = 0.352

HR, hazard ratio; CL, confidence interval; F, fixed-effect model.

Sensitivity analysis, in which each study was systematically removed, demonstrated that the pooled HRs for both DFS and PFS remained stable and robust (**Supplementary Figures S2A**, **S3A**). The Begg’s test indicated no significant publication bias for DFS (*p* = 0.107) or PFS (*p* = 0.213). However, the funnel plot for DFS pooled results was not symmetrically distributed (**Supplementary Figure S2B**, **S3B**). To address the possibility of missing studies, the trim-and-fill method was applied. The results showed that the pooled HR did not change significantly, even after accounting for potential missing studies.

### 3.5 Baseline prognostic nutritional index and objective response rate and disease control rate

**Figure 5:**
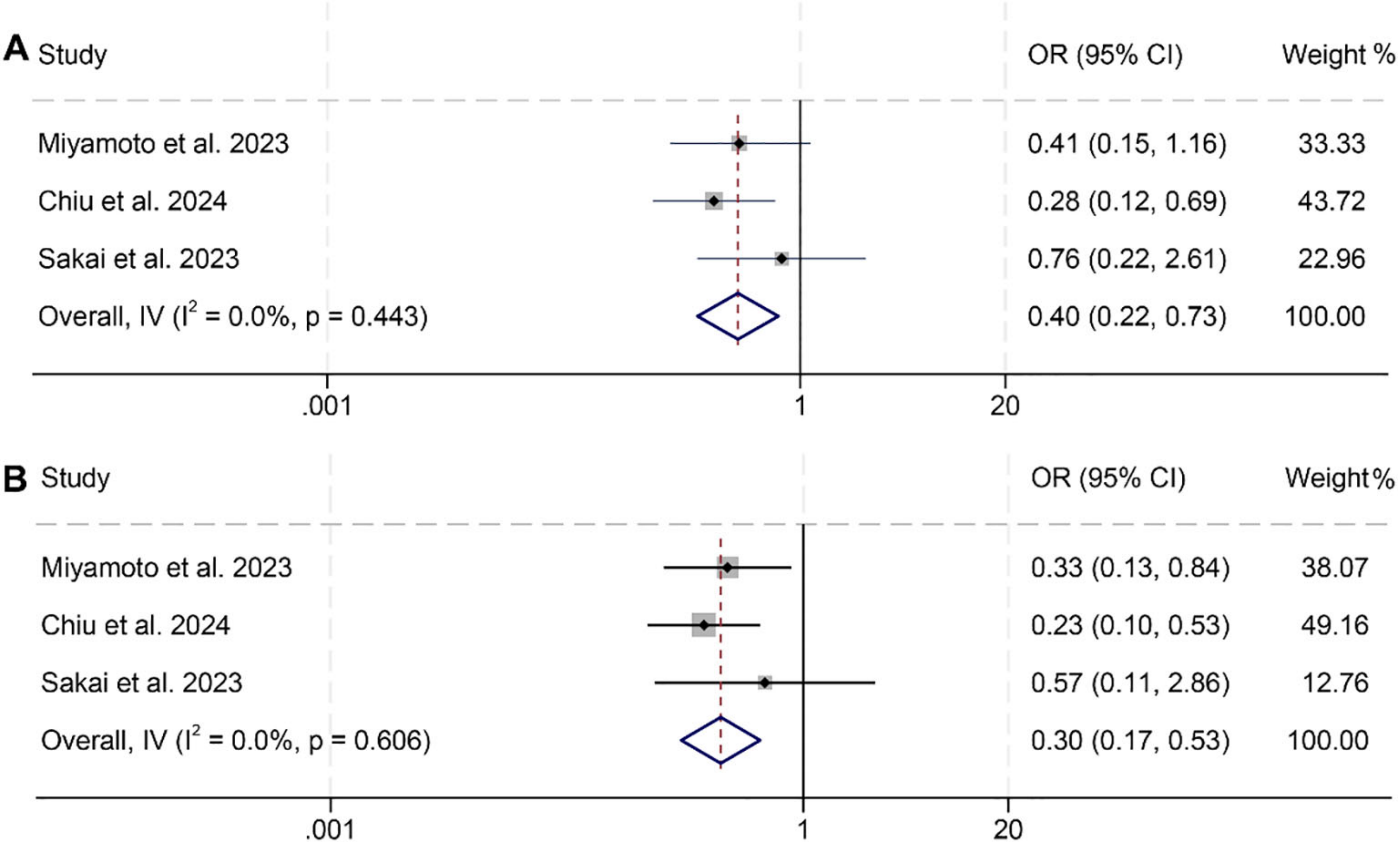
Forest plots showing the association between prognostic nutritional index (PNI) and treatment response: **(A)** Objective response rate (ORR); **(B)** Disease control rate (DCR). Both analyses were conducted using PNI as a binary variable (high vs. low).OR, odds ratio; CI, confidence interval.

We further investigated the relationship between PNI and ORR and DCR in HNSCC patients, based on three studies involving 301 individuals. Notably, no significant heterogeneity was observed across the studies (ORR, I² = 0, *p* = 0.443; DCR, I² = 0, *p* = 0.606), justifying the use of a fixed-effects model. The findings clearly indicated that patients with low PNI had a lower ORR (OR: 0.40, 95% CI: 0.22–0.73, *p* = 0.002, **Figure 5A**) and DCR (OR: 0.30, 95% CI: 0.17–0.53, *p* < 0.001, **Figure 5B**) compared to those with high PNI.

## 4 Discussion

The PNI, an inexpensive alternative to tumor markers, can be easily measured using routine preoperative blood sampling techniques and serves as a valuable prognostic tool. In this study, we found that HNSCC patients with high PNI had significantly longer survival and demonstrated a higher therapeutic response. Subgroup analyses confirmed that the association between PNI and prognosis was consistent across subgroups with different Cox models, cancer types, treatment modalities, countries, and PNI cut-off values.

There is a well-documented association between malnutrition and HNSCC (42, 43). Nutritional disorders can result from both the tumor itself and its treatments (44). Dysphagia, characterized by difficulty swallowing, may arise due to direct tumor obstruction, nerve damage, or xerostomia (45). Odynophagia, or painful swallowing, along with frequent aspiration, can lead to food aversion and recurrent pneumonia (44, 46). Additionally, reduced appetite combined with tumor-induced metabolic changes often results in catabolic energy mobilization and cachexia (47).

The PNI reflects both the nutritional and immune statuses of cancer patients. A reduced PNI indicates diminished levels of albumin and/or lymphocytes. Serum albumin serves as a marker of the body’s nutritional condition and immune functionality. Additionally, albumin supports cellular proliferation, stabilizes DNA, and acts as a biochemical buffer in metabolic reactions. It also regulates sex hormones, which may counteract cancer progression. Low serum albumin has been consistently associated with unfavorable prognoses and reduced survival rates in cancer patients (8, 22, 48).

Lymphocytes, as key components of the immune system, play a critical role in initiating antitumor responses (49). They are essential for eliminating residual tumor cells and preventing micrometastases (50, 51). Prolonged T-cell activation in cancer patients promotes tumor cell apoptosis, while tumor-infiltrating lymphocytes (TILs) present tumor-associated antigens to lymphocytes, enhancing cancer cell eradication during chemoradiotherapy. As such, lymphocytes are vital for optimizing adjuvant therapies and reducing the likelihood of tumor recurrence (52). In summary, malnutrition and lymphocytopenia may signal a persistently compromised immune system, which contributes to poorer outcomes in cancer patients.

Recent studies suggest that the prognostic value of PNI may be attributed not only to general immune competence, but also to its reflection of tumor-immune microenvironment status (40, 53, 54). Lymphocytes, particularly cytotoxic CD8χ T cells and helper CD4χ subsets, are key effectors in antitumor immunity. Low peripheral lymphocyte counts, as captured by a reduced PNI, may reflect systemic immunosuppression or immune exhaustion—both of which are associated with poor infiltration of tumor-infiltrating lymphocytes (TILs), reduced effector cytokine production, and increased expression of inhibitory receptors such as PD-1 and CTLA-4 (40, 53, 55). In this regard, PNI could indirectly reflect the immunological fitness of the host, and its capacity to mount an effective antitumor response.

Of note, a subset of the included studies focused on HNSCC patients receiving ICIs, providing a unique opportunity to explore the relevance of PNI in the context of immunotherapy (56–58). Since successful ICI response depends heavily on pre-existing immune activation and adequate T cell function, a higher PNI—indicating preserved lymphocyte-mediated immunity—may be predictive of improved responsiveness to ICIs. Conversely, low PNI levels may suggest a state of immune exhaustion or systemic inflammation, which has been associated with primary resistance to immunotherapy (56, 57). Although formal subgroup analyses on ICI-treated patients were limited due to the number of available studies, this aspect highlights the potential utility of PNI as a baseline immune fitness biomarker that could guide ICI decision-making in HNSCC.

One notable limitation of this meta-analysis is the heterogeneity in PNI cutoff values used across the included studies, which ranged from 39 to 53. This variability reflects the lack of a universally accepted threshold for defining “low” versus “high” PNI in head and neck squamous cell carcinoma (HNSCC) and complicates direct comparisons across studies. Such inconsistencies also hinder the immediate translation of findings into clinical practice, as clinicians may be uncertain which threshold to apply for risk stratification. Due to the nature of our meta-analysis, which relied on aggregate data, we were unable to perform receiver operating characteristic (ROC) curve analyses to identify an optimal cutoff. Future prospective studies using individual patient-level data are needed to determine standardized, cancer-specific PNI thresholds—ideally derived from ROC-based methods and validated across diverse populations—to enhance the clinical applicability and consistency of this biomarker.

Certain limitations of this pooled analysis should be acknowledged. First, it is worth noting that all studies included in this analysis were retrospective cohort studies, which may limit the statistical robustness of the findings. Additionally, the majority of the studies were conducted in Asia, potentially limiting the generalizability of the results to other regions. Therefore, future studies should aim to validate these findings in more diverse, multinational cohorts to ensure broader applicability of PNI as a prognostic tool in global clinical practice.

## 5 Conclusion

This study underscores the prognostic significance of the prognostic nutritional index (PNI) in predicting survival outcomes and treatment responses in patients with head and neck squamous cell carcinoma (HNSCC). Given its simplicity, cost-effectiveness, and availability from routine laboratory data, PNI may serve as a valuable adjunct in clinical decision-making. Incorporating PNI into standard prognostic assessments could aid in identifying high-risk patients, tailoring treatment intensity, optimizing nutritional support, and improving overall patient management strategies in HNSCC.

## Data Availability

The original contributions presented in the study are included in the article/**Supplementary Material**. Further inquiries can be directed to the corresponding author/s.
