# Supplementary material for: Prognostic significance of prognostic nutritional index in patients with head and neck squamous cell carcinoma
Source: Front Immunol. 2025 Aug 27;16:1597965. doi: 10.3389/fimmu.2025.1597965 (PMC12421403; doi:10.3389/fimmu.2025.1597965)
Supplement: Supplementary file 1 [file Table1.docx]

Supplementary material 1. Detailed article search strategy

((((((((((((((((((("Head And Neck Squamous Cell Carcinomas") OR ("Carcinoma, Squamous Cell of Head and Neck")) OR ("Squamous Cell Carcinoma of the Head and Neck")) OR ("Head and Neck Squamous Cell Carcinoma")) OR ("Squamous Cell Carcinoma, Head And Neck")) OR ("Oral Squamous Cell Carcinoma")) OR ("Squamous Cell Carcinoma of the Mouth")) OR ("Oral Cavity Squamous Cell Carcinoma")) OR ("Oral Squamous Cell Carcinomas")) OR ("Squamous Cell Carcinoma of the Larynx")) OR ("Squamous Cell Carcinoma of Larynx")) OR ("Laryngeal Squamous Cell Carcinoma")) OR ("Squamous Cell Carcinoma of the Nasal Cavity")) OR ("Oropharyngeal Squamous Cell Carcinoma")) OR ("Hypopharyngeal Squamous Cell Carcinoma")) OR ("Oral Tongue Squamous Cell Carcinoma")) OR (HNSCC)) OR ("Squamous Cell Carcinoma of Head and Neck"[Mesh]))) AND (((((((((((("Prognostic Nutritional Index") OR ("Index, Prognostic Nutritional")) OR ("Indices, Prognostic Nutritional")) OR ("Nutritional Index, Prognostic")) OR ("Nutritional Indices, Prognostic")) OR ("Prognostic Nutritional Indices")) OR ("Prognostic Nutritional Index")) OR ("Index, Prognostic Nutritional")) OR ("Indices, Prognostic Nutritional")) OR ("Nutritional Index, Prognostic")) OR ("Nutritional Indices, Prognostic")) OR ("Prognostic Nutritional Indices"))


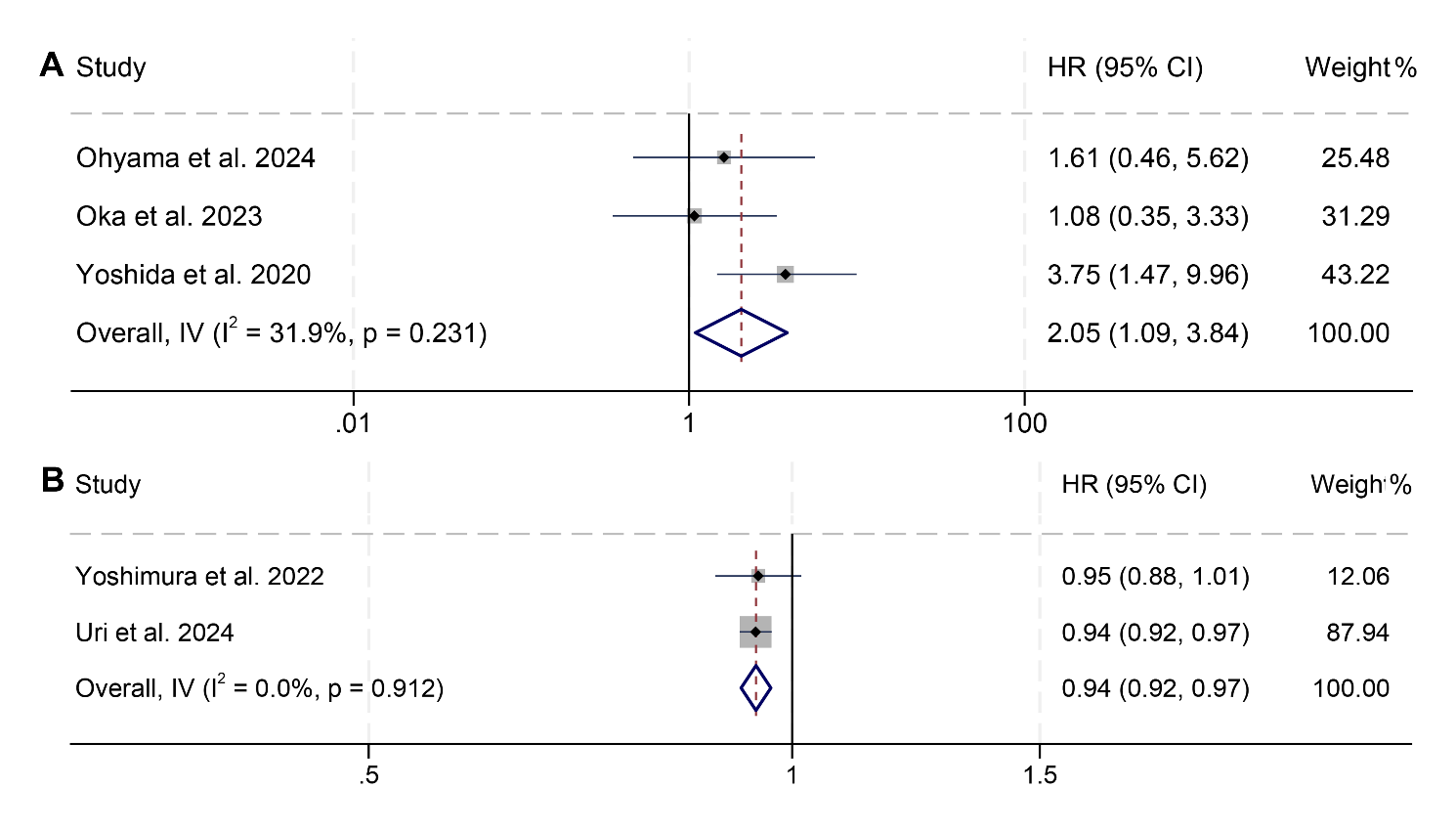


Figure S1. Forest plots of the association between prognostic nutritional index and cancer-specific survival. (A) Binary variables; (B) Continuous variables. HR, hazard ratio; CI, confidence interval.


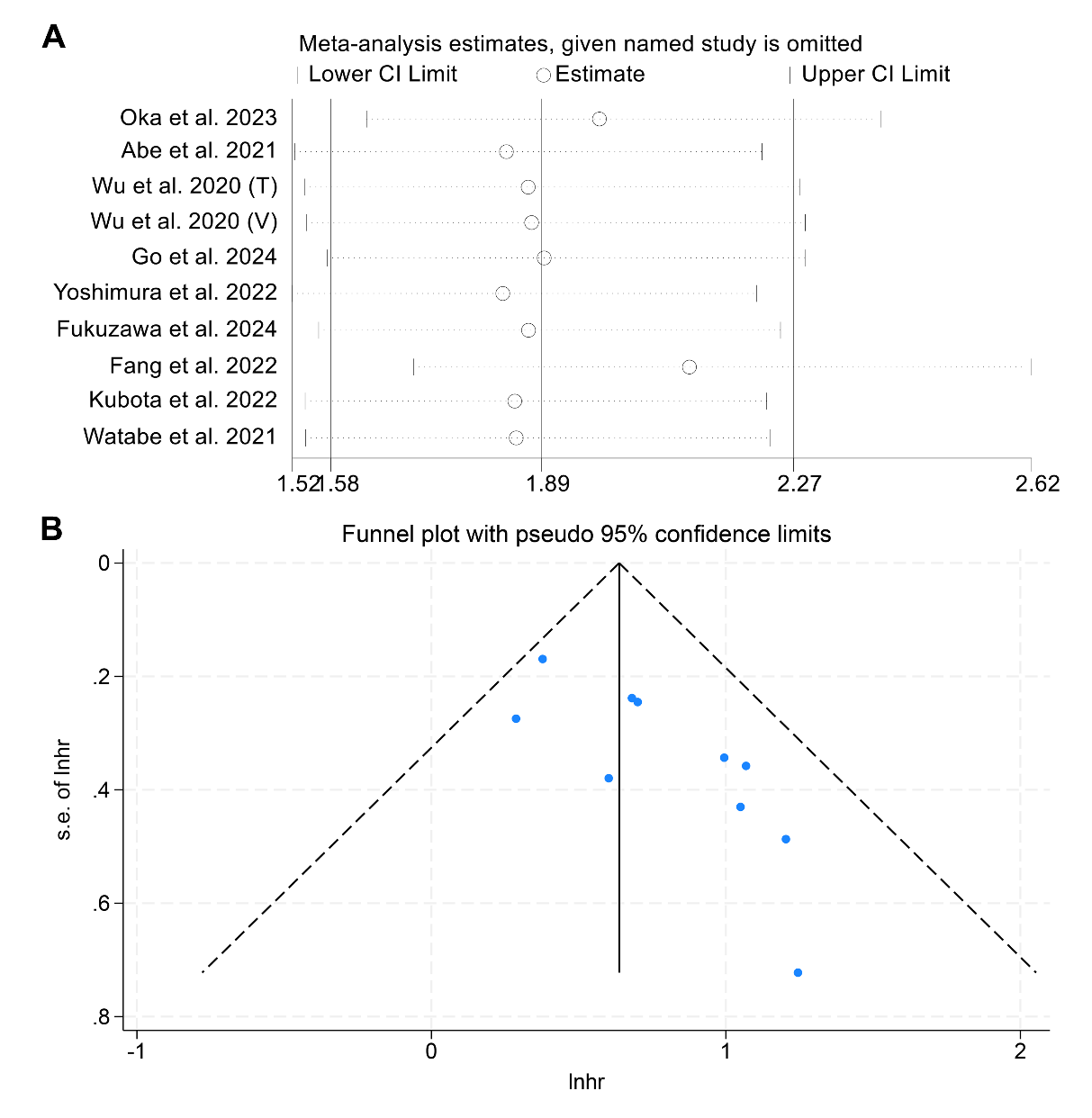


Figure S2. Sensitivity analysis of the association between prognostic nutritional index and disease-free survival (A). Funnel plots of the relationship between prognostic nutritional index and disease-free survival (B). HR, hazard ratio; CI, confidence interval.


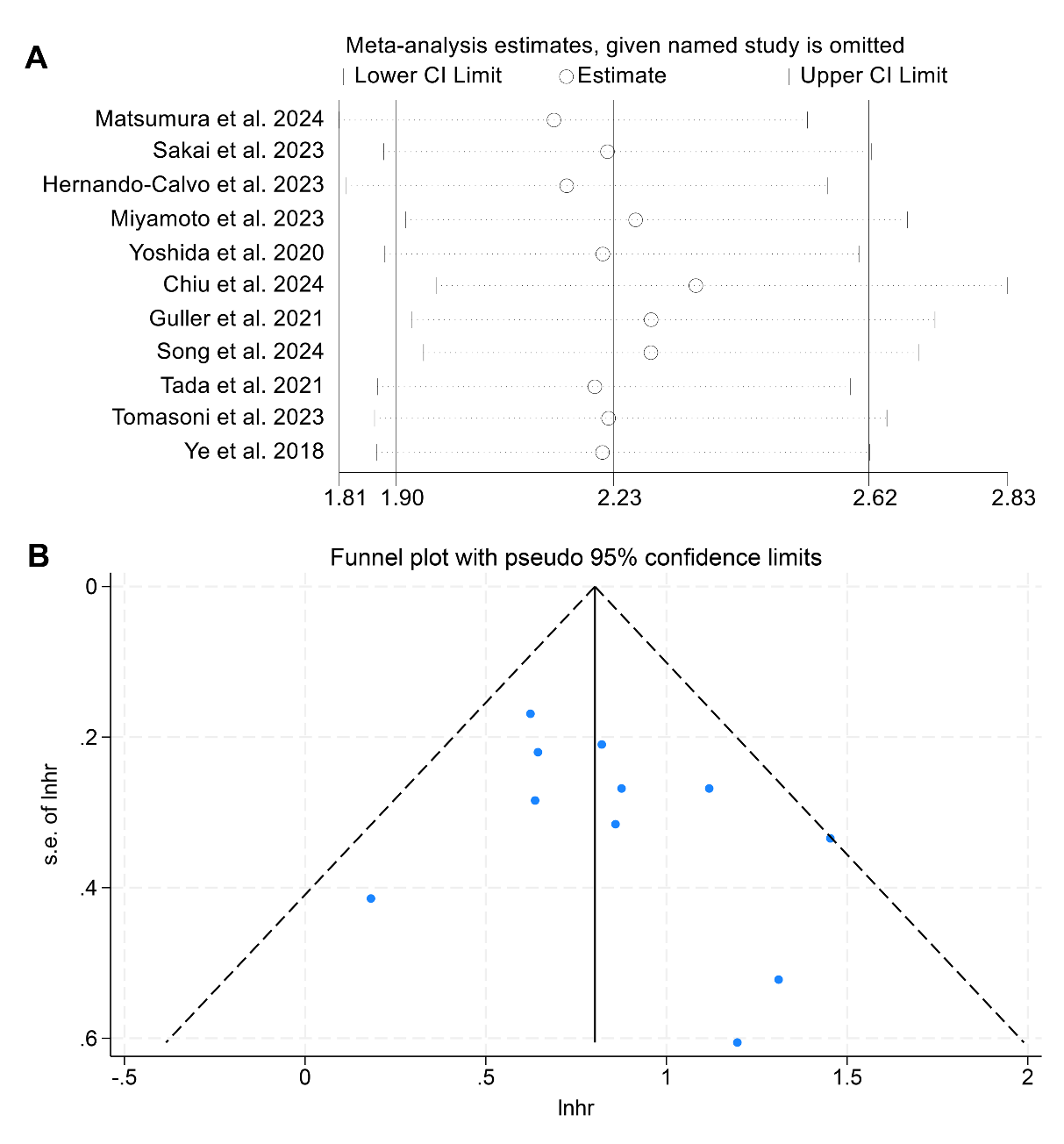


Figure S3. Sensitivity analysis of the association between prognostic nutritional index and progression-free survival (A). Funnel plots of the relationship between prognostic nutritional index and progression-free survival (B). HR, hazard ratio; CI, confidence interval.
